# Anti-latency agents to purge HIV reservoirs

**DOI:** 10.1186/1742-4690-9-S1-I16

**Published:** 2012-05-25

**Authors:** Santiago Moreno

**Affiliations:** 1 Ramón y Cajal Hospital, Madrid, Spain

## 

The persistence of latent HIV-infected cellular reservoirs represents the major hurdle to virus eradication with highly active anti-retroviral therapy (HAART), since latently infected cells remain a permanent source of viral reactivation. HIV establishes a persistent infection in CD4+ T lymphocytes (and to a lesser extent in macrophages as well), creating a persistent reservoir consisting mainly of latently infected resting memory CD4+ T cell. Although pre- and post-integration latencies have been described in HIV-1, the reservoir that appears to be the major barrier to eradication is composed of latently infected cells carrying an integrated provirus that is transcriptionally silent.

It has been suggested that reactivation of the latent reservoirs could allow effective targeting and possible eradication of the virus. Immunoactivation therapy to reduce the latent pool of HIV by treatment with the anti-CD3 antibody OKT-3 alone or in combination with interleukin-2, substantially failed to significantly decrease the viral reservoir. Non-specific T-cell activation may induce high-level viral replication above a level that can be fully contained by ART, while increasing the susceptibility of uninfected cells.

Selective targeting of HIV provirus via agents that induce the expression of quiescent HIV, but have limited effects on the uninfected host cell is an alternate approach to attack latent HIV. Activation from latency to completion of the replication cycle should result in lytic cell death of CD4+ T cells. Multiple mechanisms that contribute to the maintenance of proviral latency could be targeted to activate the latent virus. As examples of potentially useful agents, IL–7 can reactivate HIV–1 in latently infected cells *in vitro* through the induction of the Janus kinase–signal transducer and activator of transcription (JAK–STAT) signalling pathway. The use of different chemical compounds targeting the PKC signalling pathway (prostratin, bryostatin) has also been proposed as a means of reactivating viral reservoirs. Finally, HDAC blocking is an attractive potential means of inducing broad reactivation of HIV–1 reservoirs, and promising results have been achieved using the HDAC inhibitor vorinostat.

